# Molecular Epidemiology and Virulence Profiles of Colistin-Resistant *Klebsiella pneumoniae* Blood Isolates From the Hospital Agency “Ospedale dei Colli,” Naples, Italy

**DOI:** 10.3389/fmicb.2018.01463

**Published:** 2018-07-16

**Authors:** Eliana P. Esposito, Matteo Cervoni, Mariano Bernardo, Valeria Crivaro, Susanna Cuccurullo, Francesco Imperi, Raffaele Zarrilli

**Affiliations:** ^1^Department of Public Health, University of Naples “Federico II,”, Naples, Italy; ^2^Department of Biology and Biotechnology “Charles Darwin,” Sapienza University of Rome, Rome, Italy; ^3^Laboratory Affiliated to Istituto Pasteur Italia - Fondazione Cenci Bolognetti, Rome, Italy; ^4^Azienda Ospedaliera di Rilievo Nazionale (AORN) dei Colli, V. Monaldi Hospital, Naples, Italy; ^5^Centro di Ingegneria Genetica (CEINGE) Biotecnologie Avanzate, Naples, Italy

**Keywords:** mechanisms of colistin-resistance, *Klebsiella pneumonia*, blood isolates, *Galleria mellonella*, virulence profiles

## Abstract

Resistance to colistin is increasingly reported in *Klebsiella pneumoniae* clinical isolates. The aim of this study was to analyze the molecular epidemiology and virulence profiles of 25 colistin-resistant *K. pneumoniae* blood isolates from the Hospital Agency “Ospedale dei Colli,” Naples, Italy, during 2015 and 2016. Colistin MIC values of isolates ranged from 4 to 256 mg/L. The inactivation of the *mgrB* gene, encoding a negative regulator of the PhoQ/PhoP signaling system, was the most frequent mechanism of colistin resistance found in 22 out of 25 isolates. Of these, 10 isolates assigned to ST512 and PFGE types A and A4 showed identical frameshift mutation and premature termination of *mgrB* gene; 4 isolates assigned to ST258 and PFGE types A1 showed non-sense, frameshift mutation, and premature termination; 3 and 1 isolates assigned to ST258 and PFGE A2 and ST512 and PFGE A3, respectively, had insertional inactivation of *mgrB* gene due to IS*5*-like mobile element; 2 isolates assigned to ST101 and 1 to ST392 had missense mutations in the *mgrB* gene, 1 isolate assigned to ST45 showed insertional inactivation of *mgrB* gene due to IS*903*-like mobile element. *phoQ* missense mutations were found in 2 isolates assigned to ST629 and ST101, respectively, which also showed a missense mutation in *pmrA* gene. The *mcr-1-2-3-4* genes were not detected in any isolate. Colistin-resistant *K. pneumoniae* isolates showed variable virulence profiles in *Galleria mellonella* infection assays, with the infectivity of two isolates assigned to ST45 and ST629 being significantly higher than that of all other strains (*P* < 0.001). Interestingly, colistin MIC values proved to make a significant contribution at predicting lethal doses values (LD_50_ and LD_90_) of studied isolates in *G. mellonella*. Our data show that MgrB inactivation is a common mechanism of colistin resistance among *K. pneumoniae* in our clinical setting. The presence of identical mutations/insertions in isolates of the same ST and PFGE profile suggests the occurrence of clonal expansion and cross-transmission. Although virulence profiles differ among isolates irrespective of their genotypes, our results suggest that high colistin MIC could predict lower infectivity capability of the isolates.

## Introduction

The occurrence of multidrug-resistant (MDR) or extensively drug-resistant (XDR) *Klebsiella pneumoniae* infections (Bialek-Davenet et al., 2014; Bradford et al., 2015; Holt et al., 2015; Pitout et al., 2015; Cerqueira et al., 2017; Logan and Weinstein, 2017; Otter et al., 2017) has favored the use of colistin-based regimens as the most frequent therapeutic options (van Duin et al., 2013). Unfortunately, resistance to colistin has been increasingly reported in *K. pneumoniae* clinical isolates worldwide (Jeannot et al., 2017; Poirel et al., 2017).

In *K. pneumoniae*, resistance to polymixins (polymyxin B and colistin) is mainly due to modification of the lipid A phosphate moieties of the lipopolysaccharide (LPS) with a sugar or ethanolamine, which reduces the electrostatic interaction between the cationic polymixins and anionic LPS (Poirel et al., 2017). Molecular mechanisms responsible for colistin resistance rely on mutations in the genes of two component transcriptional regulatory systems PhoPQ and PmrAB, which regulate the expression of *pmrC* gene that codes for the addition of phosphoethanolamine and *pmrHFIJKLM* operon genes that encode biosynthesis and lipid A transfer of 4-amino-4-deoxy-L-arabinose (Cheng et al., 2015, 2016; Jayol et al., 2015; Wright et al., 2015; Novović et al., 2017; Poirel et al., 2017; Pragasam et al., 2017). Moreover, mutations causing loss of function in the MgrB protein, a negative feedback regulator of PhoPQ two component regulatory system (Cannatelli et al., 2013, 2014, 2016; Olaitan et al., 2014; Cheng et al., 2015; Giani et al., 2015; Poirel et al., 2015; Arena et al., 2016; Jaidane et al., 2018) and mutations in the *crrB* gene, which regulates the expression of *pmrC* gene and *pmrHFIJKLM* operon through the PmrAB two component system (Wright et al., 2015; Cheng et al., 2016; Jayol et al., 2017) have been described. Also, the acquisition of plasmid-borne *mcr 1.2* gene encoding a membrane-anchored enzyme which adds phosphoethanolamine to lipid A has been recently reported in *K. pneumoniae* (Di Pilato et al., 2016).

Worryingly, mounting evidence indicates that colistin resistance caused by inactivation of the MgrB regulator is not associated with fitness cost and decreased virulence of *K. pneumoniae* (Cannatelli et al., 2015; Arena et al., 2016) and is maintained in the absence of selective antimicrobial pressure (Cannatelli et al., 2015). Also, it has been recently demonstrated that inactivation of *mgrB* can stimulate *K. pneumoniae* virulence by decreasing the expression of antimicrobial peptides and early inflammatory response of the host (Kidd et al., 2017).

The aim of this study was to investigate the molecular epidemiology and mechanism of colistin resistance a of 25 colistin-resistant *K. pneumoniae* blood isolates from the Hospital Agency (HA) “Ospedale dei Colli,” Naples, Italy, during 2015 and 2016 and to assess the virulence profiles of *K. pneumoniae* isolates in a *Galleria mellonella* infection model.

## Materials and methods

### Setting and design of the study

The HA “Ospedale dei Colli” in Naples, Italy includes three hospitals: “V. Monaldi Hospital,” which is a 580-bed tertiary-care teaching hospital providing acute medical and surgical care within cardiology, cardiothoracic surgery, and pneumology with an active heart transplantation programme also; “D. Cotugno” Hospital, which is a 209-bed hospital and is the referral center for infectious diseases; Orthopedic Trauma Center (OTC) Hospital, which is a 143-bed hospital and is the referral center for orthopedic, neurology and neurosurgery. The hospitals are provided with 6 intensive care units (ICU)s: a neonatal ICU, a cardiac surgery ICU (CS-ICU), a post-operative ICU (PO-ICU), and a cardiorespiratory ICU (CR-ICU) in “V. Monaldi” Hospital, a medical-ICU (M-ICU) in “D. Cotugno” Hospital, and a post-operative-ICU (PO-ICU) in OTC Hospital. There is one microbiology laboratory that processes samples from all three hospitals. Surveillance of carbapenem resistant Enterobacteriaceae (CRE) in the HA “Ospedale dei Colli” was performed as previously described (Esposito et al., 2017). The present study analyzed 25 colistin-resistant *K. pneumoniae* blood isolates from 25 patients who were admitted to the HA “Ospedale dei Colli,” Naples from January 2015 to September 2016. The first colistin-resistant *K. pneumoniae* isolate from blood-culture was selected for each patient.

### Bacterial strains identification

The colistin-resistant *K. pneumoniae* strains were identified using Vitek-2 and ID-GNB card for Gram-negative bacilli according to manufacturer's instructions (bioMérieux, Marcy l'Etoile, France).

### Antimicrobial susceptibility testing

Antimicrobial susceptibilities were performed using the Vitek 2 system and the AST-GN card (bioMérieux, Marcy l'Etoile, France). Values were interpreted according to breakpoint table for interpretation of MIC values and zone diameters (European Committee on Antimicrobial Susceptibility Testing, 2016). Colistin susceptibility assay was performed according to recommendation of joint CLSI-EUCAST guidelines: http://www.eucast.org/fileadmin/src/media/PDFs/EUCAST_files/General_documents/Recommendations_for_MIC_determination_of_colistin_March_2016.pdf.

### Genotype analysis and capsular typing

Genotyping was performed using *XbaI* DNA macrorestriction, pulsed-field gel electrophoresis (PFGE) with dendrogram analysis as previously described (Del Franco et al., 2015). Multilocus sequence typing (MLST) was performed as reported in Diancourt et al. (2005) using primers and PCR conditions available at http://bigsdb.pasteur.fr/klebsiella/primers_used.html. Capsular typing was performed by PCR amplification and sequencing of *wzi* gene as previously described (Brisse et al., 2013). eBURST analysis of ST profiles and detection of carbapenemase genes was performed as described previously (Esposito et al., 2017).

### Molecular analysis of colistin resistance

The chromosomal DNA of clinical isolates was extracted using DNeasy Blood & Tissue Kit according to the manufacturer's instructions (Qiagen, Milan, Italy). Analysis of plasmid-mediated colistin resistance was performed by PCR amplification of *mcr-1, mcr-2, mcr-3*, and *mcr-4* genes as described previously (Liu et al., 2016; Xavier et al., 2016; Carattoli et al., 2017; Yin et al., 2017). Chromosomally-encoded modifications of the LPS were analyzed through amplification and sequencing of the *mgrB, pmrA, pmrB, crrB, phoP*, and *phoQ* genes as described by Cannatelli et al. (2013, 2014) and Jayol et al. (2014, 2015). The primers used are shown in Table S1. DNA sequencing of PCR products and Basic Local Alignment Search Tool (BLAST) analysis of nucleotide and deduced protein sequences were performed as previously described (Del Franco et al., 2015). Insertion sequences (ISs) were identified using the ISfinder tool (https://www-is.biotoul.fr/index.php). Complementation experiments for *mgrB* gene were performed as described previously (Cannatelli et al., 2013) with minor modification. *K. pneumoniae* transformants were selected on Mueller-Hinton agar plates supplemented with 10 mg/L of tetracycline. MIC testing of the complemented strains was performed in medium supplemented with 10 mg/L of tetracycline, to avoid plasmid loss.

### *Galleria mellonella* infection assays

*Klebsiella pneumoniae* strains were grown in MH to late exponential phase. Cells were collected by centrifugation and suspended in saline. Serial 10-fold dilutions of bacterial cell suspensions in saline were injected into *G. mellonella* larvae as described (Jander et al., 2000). Ten larvae were infected with each infecting dose and 10 larvae were injected with sterile saline as negative control. Larvae were incubated at 37°C for 3 days to monitor mortality. Each strain was tested in two or three independent experiments, with at least four dilutions injected in each experiment. Dose-dependent survival curves and lethal doses 50 and 90% (LD_50_ and LD_90_, respectively) were determined using the GraphPad Prism software as previously described (Antunes et al., 2011). For each isolate, the results obtained with the infecting doses corresponding to about 10^6^ and 10^5^ cells in the two or three independent assays were also pooled to generate Kaplan-Meier survival curves using GraphPad Prism.

### Statistical analysis

Statistical analysis of Kaplan-Meier survival curves was performed with the Log-rank (Mantel-Cox) Test using GraphPad Prism. Linear regression analysis of colistin MIC and LD_50_/LD_90_ values was performed by means of SPSS v. 20.0 (Chicago, IL, USA). *P*-values < 0.05 were considered to be statistically significant.

### Nucleotide sequence accession numbers

Nucleotide sequences of mutated *mgrB* and *phoQ* genes described in this work have been deposited in GenBank under accession numbers MG210951-MG210955 and MG214776-MG214777.

### Ethics statement

The study has been evaluated by the local Ethics committee (Comitato Etico Università degli Studi della Campania “Luigi Vanvitelli” Azienda Ospedaliera Universitaria “Luigi Vanvitelli”—HA “Ospedali dei Colli”) (protocol number 52/2018). Patients included in the study were anonymized, no written informed consent was acquired because of the retrospective nature of the study.

## Results

### Molecular epidemiology of colistin-resistant *K. pneumoniae* in the HA “ospedale dei colli,” Naples, Italy

**Figure 1:**
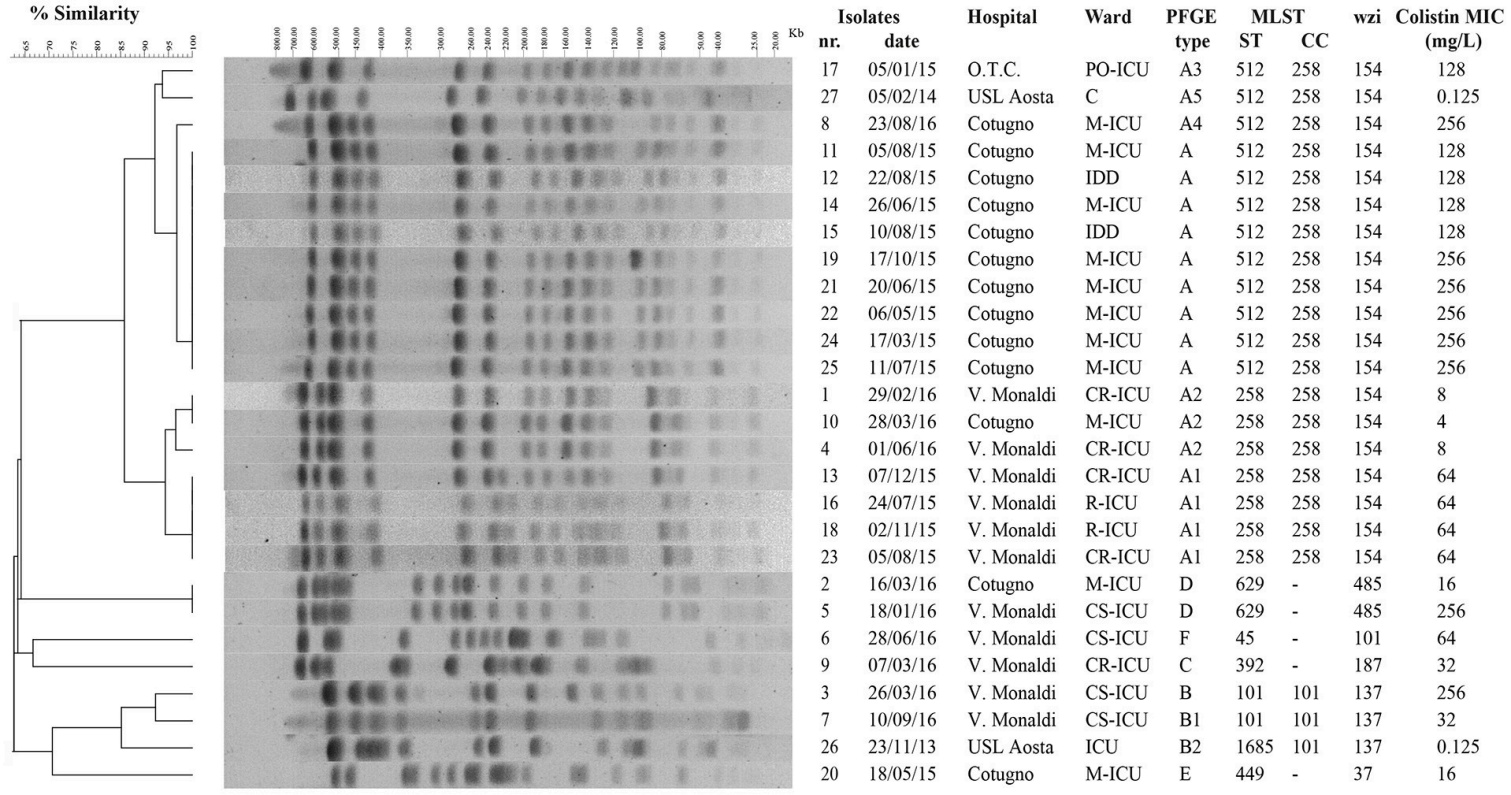
Genotypic analysis of colistin-resistant *K. pneumoniae* isolates in the HA “Ospedale dei Colli,” Naples. Dendrogram analysis of *K. pneumoniae* isolates from HA “Ospedale dei Colli,” Naples. Percentage of similarity and sizes in kilobases (kb) of lambda DNA molecular mass markers are indicated. Isolate number, isolation date, hospitals, wards, PFGE type, MLST (ST and CC), *wzi* gene, and colistin MIC values are also shown. PFGE, pulsed-field gel electrophoresis; MLST, multilocus sequence typing; ST; sequence type; CC, clonal complex.

An increase of colistin-resistant *K. pneumoniae* clinical isolates was observed in the HA “Ospedale dei Colli” during 2015 and 2016 with a prevalence of colistin-resistant *K. pneumoniae* isolates over total *K. pneumoniae* isolates of 0.15. A total of 25 isolates from blood cultures were retrospectively collected from 25 patients hospitalized in MS- ICU (11) and IDD (2) of “D. Cotugno” hospital; CR-ICU (5), CS-ICU (4), and R-ICU (2) of “V. Monaldi” hospital; PO-ICU (1) of OTC hospital (Figure 1 and Table S2). Colistin resistance was detected in initial *K. pneumoniae* isolates from 18 of the 25 patients, while 7 patients showed initial colistin-susceptible *K. pneumoniae* isolate with a subsequent colistin-resistant *K. pneumoniae* isolate. *K. pneumoniae* isolates showed a MDR phenotype. In particular, all isolate were resistant to colistin and beta-lactam/beta-lactamase inhibitor combinations (clavulanic acid/amoxicillin, piperacillin/tazobactam); all isolates but one were resistant to third and fourth generation cephems; 21 isolates were resistant to imipenem and meropenem; 22 isolates were resistant to ertapenem; all isolates but one were resistant to ciprofloxacin; 23 and 18 isolates were resistant to gentamicin and amikacin, respectively; 23 isolates were resistant to tigecycline and trimethoprim-sulfamethoxazole; 19 isolates were resistant to fosfomycin. Colistin MIC values ranged from 4 to 256 mg/L values (Table 1 and Table S2).

**Table 1:** Antimicrobial susceptibility profiles of the 25 colistin-resistant *K. pneumoniae* strains included in the study.

**Antimicrobial**	**MIC^a^** **(mg/liter)**		
	**MIC_50_**	**MIC_90_**	**Range**
Amoxicillin-clavulanic acid	>32	>32	16–>32
Piperacillin-tazobactam	>128	>128	32–>128
Ceftazidime	>64	>64	≤1–>64
Cefotaxime	>64	>64	≤1–>64
Cefepime	>64	>64	≤1–>64
Imipenem	>16	>16	≤0.25–>16
Meropenem	>16	>16	≤0.25–>16
Ertapenem	>8	>8	≤0.5–>8
Amikacin	>64	>64	≤2–>64
Gentamicin	4	>16	≤1–>16
Ciprofloxacin	>4	>4	≤0.25–>4
Tigecycline	4	>8	≤0.5–>8
Fosfomycin	128	>256	≤16–>256
Trimethoprim-sulfamethoxazole	>320	>320	≤20–>320
Colistin	128	256	4–256

a *MIC, minimal inhibitory concentration*.

To investigate whether the increase in colistin-resistant *K. pneumoniae* isolates in the HA “Ospedale dei Colli” was due to the spread of epidemic strains, the 25 colistin-resistant *K. pneumoniae* isolates from the HA “Ospedale dei Colli” and 2 colistin-susceptible *K. pneumoniae* reference isolates were genotyped (isolates 8 and 17 in Del Franco et al., 2015). Molecular typing using PFGE and dendrogram analysis identified 6 types, which we named from A to F, which differed in migration of more than 6 DNA fragments and showed a similarity of < 80% at dendrogram analysis. PFGE types A and B could be further classified into 5 (A1–A5) and 2 (B1–B2) subtypes, respectively, which showed one-fragment to five-fragment variation in the macro-restriction pattern and a similarity of >80% at dendrogram analysis. Of 25 colistin-resistant *K. pneumoniae* isolates, 9 isolates showed PFGE type A; 4, 3, 1, and 1 isolates showed PFGE types A1, A2, A3, and A4, respectively; 2 isolates showed PFGE types B and B1; 2 isolates PFGE type D; three sporadic isolates showed PFGE types C, E, or F. The two colistin-susceptible isolates were assigned to PFGE types A5 and B2 (Figure 1). MLST analysis assigned PFGE types A, A3, A4, and A5 to ST512; PFGE types A1 and A2 to ST258; PFGE types B and B1 to ST101, PFGE type B2 to ST1685; PFGE type C to ST392; PFGE type D to ST629; PFGE types E and F to ST449 and ST45, respectively. As ST512 and ST1685 represent single locus variants of ST258 and ST101, respectively, eBURST analysis clustered ST258 and ST512 in clonal complex (CC) 258, while ST101 and ST1685 in CC101 (Figure 1). Capsular typing identified *wzi* allele 154 for isolates assigned to ST258 and ST512, wzi allele 137 for isolates assigned to ST101 and ST1685, wzi allele 485 for isolates assigned to ST629, wzi alleles 37, 101, and 187 for isolates assigned to ST449, ST101, and ST392, respectively (Figure 1). The screening for carbapenemases revealed that all colistin-resistant isolates belonging to CC258 (ST512 and ST258) and isolates assigned to ST101/B and ST392/C were KPC-3 producers, while the isolate assigned to ST101/B1 and one isolate assigned to ST629/D were VIM-1 producers. No carbapenemase genes were found in isolates assigned to ST445/E and ST45/F and in one of the isolates assigned to ST629/D (Table 2).

**Table 2:** Molecular mechanisms of colistin-resistance in 25 *K. pneumoniae* blood isolates from HA “Ospedale dei Colli,” Naples.

**Strain**	**Genotype**	**Carbapenemase**	**Colistin MIC^a^**	***mgrB*^b^**	**PhoQ^c^**	**CrrB^d^**	**PmrA^c^**	**PmrB^c^**
1	ST258/A2	KPC-3	8	Insertional inactivation, IS5-like element at nt 75 (FW)		WT		
2	ST629/D	VIM-1	16	WT	D150G^e^	L296Q^f^	WT	R256G^e^
3	ST101/B	KPC-3	256	t95g (V32G)	D150G^e^ W215G	–	A217V	WT
4	ST258/A2	KPC-3	8	Insertional inactivation, IS5-like element at nt 75 (FW)		WT		
5	ST629/D	–	256	WT	D150G^e^ L257P	Q287K L296Q^f^	WT	WT
6	ST45/F	–	64	Insertional inactivation, IS903 element at nt 69 (FW)		–		
7	ST101/B1	VIM-1	32	t50g (L17R)		–		
8	ST512/A4	KPC-3	256	Δg19 (frameshift mutation)		WT		
9	ST392/C	KPC-3	32	t139a (W47R)		L296Q^f^		
10	ST258/A2	KPC-3	4	Insertional inactivation, IS5-like element at nt 75 (FW)		WT		
11	ST512/A	KPC-3	128	Δg19 (frameshift mutation)		WT		
12	ST512/A	KPC-3	128	Δg19 (frameshift mutation)		WT		
13	ST258/A1	KPC-3	64	c88t (non-sense, premature termination)		WT		
14	ST512/A	KPC-3	128	Δg19 (frameshift mutation)		WT		
15	ST512/A	KPC-3	128	Δg19 (frameshift mutation)		WT		
16	ST258/A1	KPC-3	64	c88t (non-sense, premature termination)		WT		
17	ST512/A3	KPC-3	128	Insertional inactivation, IS5-like element at nt 75 (FW)		WT		
18	ST258/A1	KPC-3	64	c88t (non-sense, premature termination)		WT		
19	ST512/A	KPC-3	256	Δg19 (frameshift mutation)		WT		
20	ST449/E	–	16	WT	D150G^e^	–	WT	WT
21	ST512/A	KPC-3	256	Δg19 (frameshift mutation)		WT		
22	ST512/A	KPC-3	256	Δg19 (frameshift mutation)		WT		
23	ST258/A1	KPC-3	64	c88t (non-sense, premature termination)		WT		
24	ST512/A	KPC-3	256	Δg19 (frameshift mutation)		WT		
25	ST512/A	KPC-3	256	Δg19 (frameshift mutation)		WT		

a *Colistin MICs are expressed as mg/L*.

b *Nucleotide (nt) numbers indicate the positions of mutations or of the insertion sites of IS (insertion sequence)s; numbering refers to the coding sequence of the mgrB open reading frame (ORF) of the colistin-susceptible K. pneumoniae KKBO-1 strain (GenBank accession no. AVFC00000000.1), considering number 1 as the first base of the GTG start codon; amino acid changes in the deduced protein sequence are indicated in parenthesis. FW indicates that the transposase gene is in the same orientation as the mgrB gene. Wild type (WT) indicates that the sequence of the mgrB ORF was identical to that of the colistin-susceptible K. pneumoniae KKBO-1 strain (Cannatelli et al., 2013). Δ, deletion*.

c *Positions and amino acid substitutions in PhoQ, PmrA and PmrB deduced protein sequences respect to colistin-susceptible K. pneumoniae NUTH-K2044 strain (GenBank accession no. AP006725.1) (Cheng et al., 2015) are shown*.

d *Positions and amino acid substitutions in the CrrB protein sequence with respect to the CrrB protein of the colistin-susceptible K. pneumoniae UHKPC27 strain (GenBank accession no. APVR00000000.1) (Wright et al., 2015) are shown; the (–) symbol indicates that the crrB gene was not amplified with the primers used in this work*.

e *Mutations that do not affect MIC of the colistin susceptible K. pneumoniae NUTH- K2044 strain (Cheng et al., 2015)*.

f *Mutation previously found in the colistin susceptible K. pneumoniae XH209 strain (Hua et al., 2014)*.

Based on PFGE and MLST typing data, four distinct epidemic genotypes were identified in 2 or more than 2 colistin-resistant *K. pneumoniae* isolates from patients in the HA “Ospedale dei Colli,” which we named ST512/A, ST258/A1, ST512/A2, and ST629/D. Molecular epidemiology of colistin-resistant *K. pneumoniae* in different wards of the HA “Ospedale dei Colli” showed that ST512/A genotype was isolated in 7 and 2 patients from M-ICU and IDD wards of “D. Cotugno” hospital during 2015; ST258/A1 and ST258/A2 genotypes were isolated in 2, 2, and 2 patients from CR-ICU, R-ICU, and R-ICU wards of “V. Monaldi” hospital during 2015 and 2016, respectively, and 1 patients from M-ICU of “D. Cotugno” hospital during 2016; ST629/D genotype was isolated in 1 from M-ICU ward of “D. Cotugno” hospital and 1 patient from CS-ICU ward of “V. Monaldi” hospital during 2016. Genotypes ST512/A3, ST512/A4, ST101/B, ST101/B1 ST392/C, ST/449/E, and ST45/F were isolated from single patients in different wards and were considered as sporadic (Figure 1 and Figure S1).

### Molecular mechanisms of colistin-resistance in *K. pneumoniae* blood isolates from the HA “ospedale dei colli,” Naples

In order to analyze the molecular mechanisms responsible for colistin-resistance, the presence of *mcr* genes encoding membrane-anchored enzymes which add phosphoethanolamine to lipid A was investigated in all isolates. Because the *mcr-1, mcr-2, mcr-3*, and *mcr-4* genes were not detected in any isolate, the presence of mutations in regulators of PhoQ/PhoP and PmrA/PmrB signaling systems was investigated.

Mutations in the *mgrB* gene, which encodes a negative regulator of the PhoQ/PhoP signaling system, were present in 22 out of 25 isolates. In particular, identical small deletion Δg19, which causes frameshift mutation and premature termination of MgrB (GenBank accession no. MG210954), was found in 9 and 1 isolates assigned to ST512/A and ST512/A4 genotypes, respectively; non-sense mutation c88t, which caused premature termination of MgrB, was found in 4 isolates assigned to ST258/A1 genotype (GenBank accession no. MG210955); missense mutations V32G (NCBI Reference Sequence: WP_094312677.1), L17R (GenBank accession no. MG210952), and W47R (GenBank accession no. MG210953) were found in isolates assigned to ST101/B, ST101/B1, and ST392/C genotypes, respectively (Table 2). The insertional inactivation of the *mgrB* gene was detected in 5 isolates. IS*5*-like mobile element at nt 75 of *mgrB* gene (GenBank accession no. MG214776) was found in 3 and 1 isolates assigned to ST258/A2 and ST512/A3 genotypes, respectively. An IS903-like element (97% identity to IS903) at nucleotide 69 of *mgrB* gene (Genbank accession no. MG214777) was found in 1 isolate assigned to ST45/F genotype. Two isolates assigned to ST629/D and 1 isolate assigned to ST449/E carried a wild type *mgrB* gene (Table 2).

Missense mutations were also found in *pmrA, pmrB, phoQ*, and *crrB* genes when compared to the genes present in colistin-susceptible *K. pneumoniae* NUTH-K2044 strain (GenBank accession no. AP006725.1) (Cheng et al., 2015). For PhoQ deduced protein sequences, a common mutation D150G was found in isolates assigned to ST629/D, ST101B-B1, and ST449/E genotypes. This mutation has been previously described and was not related with colistin resistance (Cheng et al., 2015). The substitutions W215G (Genbank accession no. MG210951) and L257P (NCBI Reference Sequence: WP_087760419.1) were also found in isolates assigned to ST101/B and ST629/D genotypes; however, no information is available about their implication in colistin resistance. In *pmrA*, a mutation leading to the substitution A217V was found in the isolate assigned to ST101/B genotype. This mutation has been already reported in *K. pneumoniae* (NCBI Reference Sequence: WP_032419166.1), but there is no information regarding colistin-susceptibility of the isolates. Finally, a mutation causing the substitution R256G in PmrB, which has been previously demonstrated to not confer colistin resistance (Cheng et al., 2015), was found in 1 isolate assigned to ST629/D genotype.

The sequence of the signal-transducing histidine kinase of the two-component regulatory system CrrAB was also investigated. In accordance with previous data (Wright et al., 2015), the *crrB* gene was found in 21 out of 25 isolates. Of these, 18 isolates assigned to ST512/A-A3-A4 and ST258/A1-A2 genotypes had CrrB identical to that of the colistin-susceptible *K. pneumoniae* UHKPC27 strain (GenBank accession no. APVR00000000.1) (Wright et al., 2015), while 2 and 1 isolates assigned to ST629/D and ST392/C genotypes, respectively, carried a missense mutation leading to L296Q substitution in the CrrB protein sequence. This CrrB substitution has been described already in colistin-susceptible *K. pneumoniae* XH209 strain (Hua et al., 2014), suggesting that is not responsible for colistin resistance of our isolates.

The above data indicate that MgrB inactivation was the most common mechanism of colistin resistance among *K. pneumoniae* isolates. In support of this, complementation experiments demonstrated that plasmid pACYC-*mgrB*, carrying WT *mgrB* gene fused to its own promoter, but not the empty plasmid pACYC184, was able to restore colistin susceptibility in selected *K. pneumoniae* isolates showing either insertional inactivation (isolates 1 and 17) or different mutations (isolates 3, 7, 16, and 22) in the *mgrB* gene (Table 3).

**Table 3:** Colistin MICs of selected *K. pneumoniae* isolates carrying either the pACYC184 or the pACYC-*mgrB* plasmid.

**Strain**	***mgrB* mutation**	**Colistin MIC (mg/L)^a^**	
		**+ pACYC184**	**+ pACYC-*mgrB***
1	Insertional inactivation, IS5-like element at nt 75 (FW)	8	0.5
3	t95g (V32G)	256	1
7	t50g (L17R)	32	0.5
16	c88t (premature termination)	64	0.5
17	Insertional inactivation, IS5-like element at nt 75 (FW)	128	0.25
22	Δg19 (frameshift mutation)	128	0.5

a *MIC assays were performed using Mueller-Hinton Broth II supplemented with 10 mg/L of tetracycline*.

### Virulence profiles of colistin-resistant *K. pneumoniae* isolates

In order to verify the effect of colistin resistance on pathogenicity, we assessed the virulence profile of 16 colistin resistant *K. pneumoniae* isolates, representative of all different genotypes identified in our collection and of different levels of colistin resistance for each genotype, and of the 2 colistin sensitive strains in the insect *G. mellonella*, which has been extensively used as an infection model to investigate the pathogenic potential of *K. pneumoniae* strains (Insua et al., 2013; McLaughlin et al., 2014; Arena et al., 2016).

**Figure 2:**
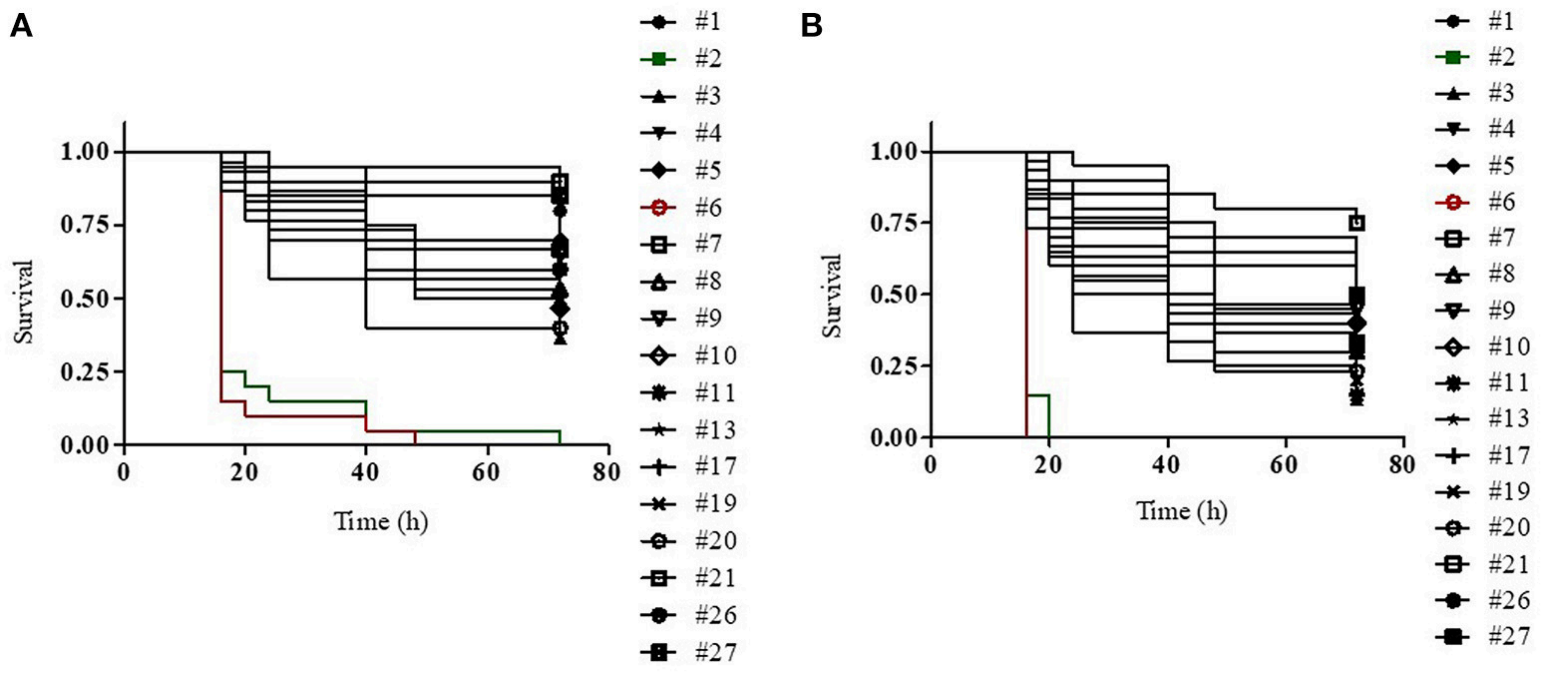
Kaplan–Meier survival curves of *G. mellonella* larvae infected with **(A)** 1 (±0.26) × 10^5^ or **(B)** 1 (±0.26) × 10^6^ viable cells of the indicated *K. pneumoniae* strains. For each strain, 20 or 30 larvae were infected in two or three independent assays, respectively. In both experiments, the infectivity of isolates 2 and 6 (green and red lines, respectively) is significantly higher than that of all other strains (*P* < 0.001).

We generated dose-dependent survival curves for all isolates, which allowed to determine their LD_50_ and LD_90_ values in *G. mellonella* larvae (Table 4). High variability in the infectivity of the different isolates in *G. mellonella* was observed, with LD_90_ values ranging from few thousands of cells (isolate 6) to more than 40 millions of cells (isolate 21) (Table 4). This huge variability is however mainly related to the presence of a couple of hyper-virulent strains (isolates 6 and 2), characterized by LD_90_ values more than 100- or 10-fold lower than those of all other isolates respectively, and an isolate (21) which showed very low infectivity, with an LD_90_ value >5-fold higher than those of all other isolates (Table 4). The median LD_50_ and LD_90_ values for the remaining isolates were about 1.5 × 10^5^ and 5 × 10^6^ cells respectively, in line with previous reports on the pathogenicity of different *K. pneumoniae* strains in *G. mellonella* larvae (Insua et al., 2013; Wand et al., 2017). The higher infectivity of the isolates 2 and 6 was also confirmed by Kaplan-Maier survival curves generated with two different infecting doses (about 10^6^ and 10^5^ cells; Figure 2 and Figure S2). Indeed, larvae infected with these isolates died much faster as compared to those infected with other *K. pneumoniae* isolates, with survival curves at both infecting doses significantly different from those obtained with all other strains (*P* < 0.001) (Figure 2 and Figure S2). A relationship was found between LD_50_ and LD_90_ values and colistin MIC values among isolates, those with elevated colistin MIC values having higher LD_50_ and LD_90_ values (Table 4). Although correlation coefficients were not significant, simple regression analysis showed that colistin MIC values make a significant contribution at predicting both LD_50_ and LD_90_ values, though the latter appears to be more affected. In detail, colistin MIC accounts for 27.9% (F value 6.183, Sig. 0.024) and 42.7% (F value 11.937, Sig. 0.003) of LD_50_ and LD_90_ variations, respectively.

**Table 4:** Lethal doses 50% (LD_50_) and 90% (LD_90_) in *G. mellonella* larvae for the indicated *K. pneumoniae* strains^a^.

**Strain^b^**	**Genotype^c^**	**Colistin MIC^d^**	**LD_50_**	**LD_90_**	***R*^2^**
6	ST45/F	64	5.98 × 10^2^	2.21 × 10^3^	0.988
2	ST629/D	16	8.56 × 10^3^	3.51 × 10^4^	0.964
3	ST101/B	256	1.98 × 10^4^	4.84 × 10^5^	0.807
8	ST512/A4	256	6.51 × 10^4^	4.83 × 10^6^	0.768
4	ST258/A2	8	7.18 × 10^4^	1.71 × 10^6^	0.659
10	ST258/A2	4	7.46 × 10^4^	2.58 × 10^6^	0.740
11	ST512/A	128	9.45 × 10^4^	2.21 × 10^6^	0.825
20	ST449/E	16	9.93 × 10^4^	3.43 × 10^6^	0.832
17	ST512/A3	128	1.02 × 10^5^	3.57 × 10^6^	0.822
19	ST512/A	256	1.35 × 10^5^	2.82 × 10^6^	0.749
26	ST1685/B2	0.125	2.29 × 10^5^	4.99 × 10^6^	0.702
7	ST101/B1	32	4.46 × 10^5^	6.03 × 10^6^	0.790
9	ST392/C	32	7.24 × 10^5^	8.27 × 10^6^	0.824
5	ST629/D	256	7.86 × 10^5^	6.42 × 10^6^	0.997
27	ST512/A5	0.125	8.46 × 10^5^	5.32 × 10^6^	0.970
1	ST258/A2	8	8.80 × 10^5^	6.58 × 10^6^	0.933
13	ST258/A1	64	1.01 × 10^6^	7.58 × 10^6^	0.940
21	ST512/A	256	2.91 × 10^6^	4.24 × 10^7^	0.893

a *LD_50_, LD_90_, and R^2^-values were determined using the GraphPad Prism software and the dose-dependent survival curves shown in the Figure S3. LD_50_ and LD_90_ are expressed as number of viable cells*.

b *Strains have been ordered according to increasing LD_50_ values*.

c *ST and PFGE profiles are shown*.

d *Colistin MICs are expressed as mg/L*.

## Discussion

In the present study, we analyzed the molecular epidemiology, mechanism of resistance, and virulence profiles of colistin-resistant *K. pneumoniae* blood isolates from HA “Ospedale dei Colli,” Naples. Our data demonstrate the cross transmission and clonal expansion of 4 epidemic genotypes in different patients and wards: ST512/A genotype was isolated in M-ICU and IDD wards of “D. Cotugno” hospital, ST258/A1 and ST258/A2 genotypes were isolated in CR-ICU, R-ICU and R-ICU wards of “V. Monaldi” hospital and M-ICU of “D. Cotugno,” ST629/D genotype in M-ICU ward of “D. Cotugno” hospital and CS-ICU ward of “V. Monaldi” hospital. This is in agreement with previous data reporting clonal expansion of *K. pneumoniae* isolates assigned to CC258 in Italy and world-wide (Bialek-Davenet et al., 2014; Gaiarsa et al., 2015; Giani et al., 2015; Conte et al., 2016; Cerqueira et al., 2017). Of the 7 *K. pneumoniae* sporadic isolates, 2 were assigned to the emerging epidemic clonal lineage CC101 (Del Franco et al., 2015; Conte et al., 2016; Novović et al., 2017; Jaidane et al., 2018). In agreement with previous data (Cannatelli et al., 2014; Jayol et al., 2014; Bradford et al., 2015; Giani et al., 2015; Wright et al., 2015), 20 out of 25 colistin-resistant *K. pneumoniae* isolates were carbapenem-resistant and KPC-3 producing, while 2 were VIM-1 producing isolates.

Colistin-resistant *K. pneumoniae* was the initial isolate from 18 patients (72%), while isolates were initially colistin-susceptible and subsequently colistin-resistant in 7 patients (28%). The inactivation of MgrB, the negative regulator of the PhoQ/PhoP signaling system, was found in 22 (88%) colistin-resistant *K. pneumoniae* isolates from HA “Ospedale dei Colli” and was caused by 7 different mechanisms of *mgrB* alterations. This is consistent with a recent study showing that the emergence of colistin-resistant *K. pneumoniae* in two England Hospitals was due to the transmission among patients of two distinct genotypes harboring three distinct mechanisms of colistin resistance (Otter et al., 2017). In agreement with previous data (Cannatelli et al., 2014; Olaitan et al., 2014; Giani et al., 2015; Poirel et al., 2015, 2017; Wright et al., 2015; Jaidane et al., 2018), a truncated MgrB was generated by one single nucleotide deletion, which causes frameshift mutation and premature termination in 10 isolates assigned to ST512/A and ST512/A4 genotypes, or by non-sense mutation c88t, which causes premature termination in 4 isolates assigned to ST512/A1. Mutated MgrB was generated by 3 distinct mutations in 3 isolates assigned to ST101/B, ST101/B1 and ST392/C genotypes. The insertional inactivation of *mgrB* gene due to IS*5*-like or IS-903-like mobile elements was observed in 2, 1, and 1 isolates assigned to ST512/A2, ST512/A3, and ST45/F, respectively, similarly to data reported in Cannatelli et al. (2013), Olaitan et al. (2014), and Poirel et al. (2015). In particular, the insertion of IS*5*-like mobile element at nt 75 of *mgrB* gene was in the same position to that found in *K. pneumoniae* KKBO-4 strain (Cannatelli et al., 2013), suggesting the existence of a specific hot spot for IS*5* insertion in the *mgrB* gene. The presence of identical *mgrB* alterations in isolates from the same ward/hospital assigned to the same ST and PFGE profile demonstrates the occurrence of clonal expansion and cross-transmission of the colistin-resistant isolates within hospitals. Allelic variants with respect to the genes present in a colistin-susceptible *K. pneumoniae* reference strain were also found for *pmrA, pmrB, phoQ*, and *crrB* genes in some *K. pneumoniae* isolates described in this study; these variants have been already described in the literature and proposed to be not related with colistin resistance.

The above all data suggest that colistin-resistance in *K. pneumoniae* isolates was caused by alterations identified in the *mgrB* gene. In support of this, complementation with a wild type *mgrB* allele successfully restored colistin susceptibility in isolates having mutations or insertional inactivation of *mgrB* gene (Table 3). In particular, colistin susceptibility was restored also in isolate 3, which carries mutations in both *mgrB* and *phoQ* genes, further confirming that resistance was mainly caused by inactivation of MgrB. Notably, three isolates showed a wild type *mgrB* gene and no mutations in the other colistin-resistance related genes here analyzed, suggesting the presence of still-unidentified mechanism(s) alternative to mutations of *mgrB* gene.

We also demonstrate that colistin-resistant *K. pneumoniae* blood isolates from HA “Ospedale dei Colli” showed high variability in the infectivity in *G. mellonella* larvae, with up to 10-fold differences in lethal dose values among isolates assigned to the same ST and PFGE genotype and two hyper-virulent isolates assigned to ST45 and ST629 having LD_90_ values more than 100- or 10-fold lower than those of all other isolates, respectively. This is consistent with previous studies showing that *K. pneumoniae* has a high genome variability and a large accessory genome, which includes virulence functions associated with invasive disease in humans and antimicrobial resistance genes associated with hospital-acquired infections (Bialek-Davenet et al., 2014; Holt et al., 2015; Cerqueira et al., 2017). *K. pneumoniae* isolates assigned to CC258 and to other STs associated with hospital-acquired infections have acquired antimicrobial resistance genes and are usually devoid of virulence genes (Bialek-Davenet et al., 2014; Cerqueira et al., 2017), although the acquisition of yersiniabactin has been observed in many isolates of the epidemic KPC-producing CC258 (Holt et al., 2015). In further agreement with our data, carbapenem-resistant *K. pneumoniae* ST258 isolates associated with nosocomial infections exhibit variability in the infectivity of *G. mellonella* and in other virulence-associated traits (Diago-Navarro et al., 2014). Our data also showed no significant reduction in the infectivity in colistin-resistant isolates assigned to ST101 or ST258/ST512 genotypes with inactivation of the *mgrB* gene as compared to their respective colistin susceptible isolates (26 and 27; Figure 2). This is an agreement with previous studies showing that colistin resistance caused by the inactivation of the MgrB regulator is not associated with fitness cost and decreased virulence of ST258 KPC carbapenemase-producing *K. pneumoniae* (Cannatelli et al., 2015; Arena et al., 2016). Our data are also in partial agreement with a recent study showing that inactivation of MgrB actually enhanced virulence of *K. pneumoniae* Kp52145 strain, serotype O1:K2, belonging to the virulent CC65 (Kidd et al., 2017), although it should be considered that, even when they belong to the same ST and PFGE type, our clinical isolates are not isogenic, and thus a direct causal relationship between specific mutations and infectivity cannot be inferred. In this regard, it is worth noting that another study indeed reported that colistin-resistant *K. pneumoniae* strains carrying distinct mutations in *pmrB* or *mgrB* genes were highly variable in their pathogenicity in the *G. mellonella* infection model (Wand et al., 2017). Overall, all the above data strongly suggest that the pathogenicity of *K. pneumoniae* isolates is independent of specific colistin-resistance mechanisms and might be influenced by variability in the genetic background of the different strains.

Finally, it is interesting to note that some isolates assigned to the same genotype, carrying the same mutation in *mgrB* and showing identical alleles for all the other colistin-resistance related genes here investigated, showed variability in colistin MIC values (Table 2), indicating that other, still-unidentified genetic mechanisms likely contribute to the acquisition of high levels of colistin resistance in *mgrB* defective backgrounds. Notably, although we did not observe any significant correlation between colistin-resistance mechanisms and virulence, in line with previous reports (Wand et al., 2017), our data suggest that the level of colistin MIC of *K. pneumoniae* isolates is predictive of their lethality (LD_50_ and LD_90_ values) in *G. mellonella*. Indeed, in our experimental setting high colistin MIC values are predictive of lower virulence of the isolates, suggesting that genetic adaption to high levels of colistin resistance could somehow impair *K. pneumoniae* infectivity. Further studies using larger collections of isolates, isogenic *in vitro* evolved colistin-resistant mutants and/or different infection models are however required to verify this hypothesis. Moreover, as whole genome sequencing has not been performed in this study, at this stage we cannot rule out that the acquisition of specific virulence-related gene(s) could partly account for the different pathogenic behavior of our isolates.

## Author contributions

FI and RZ conceived the study and participated in its design and coordination. MB, SC, and VC collected the microbiological and epidemiological data. EE, MC, and MB performed laboratory analyses. FI, VC, and RZ performed data analyses. EE, FI, and RZ wrote the manuscript. All authors read and approved the final manuscript.

### Conflict of interest statement

The authors declare that the research was conducted in the absence of any commercial or financial relationships that could be construed as a potential conflict of interest.

## Supplementary material

Figure S1 Circulation of colistin-resistant *K. pneumoniae* genotypes in the HA “Ospedale dei Colli” from January 2015 to September 2016. The number of isolates and wards are shown.

Figure S2 Kaplan–Meier survival curves of *G. mellonella* larvae infected with 1 (±0.26) × 10^6^ (continuous line) or 1 (±0.26) × 10^5^ (dashed line) viable cells of the indicated *K. pneumoniae* strains. For each strain, 20 or 30 larvae were infected in two or three independent assays, respectively. In both experiments, the infectivity of strains #2 and 6 is significantly higher than that of all other strains (*P* < 0.001).

Figure S3 Dose-dependent survival curves, generated by the GraphPad Prism software, of *G. mellonella* larvae infected with different doses of the *K. pneumoniae* strains #1, 2, 3, 4, 5, 6, 7, 8, 9, 10, 11, 13, 17, 19, 20, 21, 26, and 27. These curves have been used to retrieve the LD50, LD90, and *R*^2^ values reported in Table 4.

Table S1 Oligonucleotides used in the study.

Table S2 Phenotypic and genotypic features of *K. pneumoniae* isolates included in the study.
