# Supplementary figures and images for: Molecular Epidemiology and Virulence Profiles of Colistin-Resistant Klebsiella pneumoniae Blood Isolates From the Hospital Agency “Ospedale dei Colli,” Naples, Italy
Source: Front Microbiol. 2018 Jul 16;9:1463. doi: 10.3389/fmicb.2018.01463 (PMC6054975; doi:10.3389/fmicb.2018.01463)

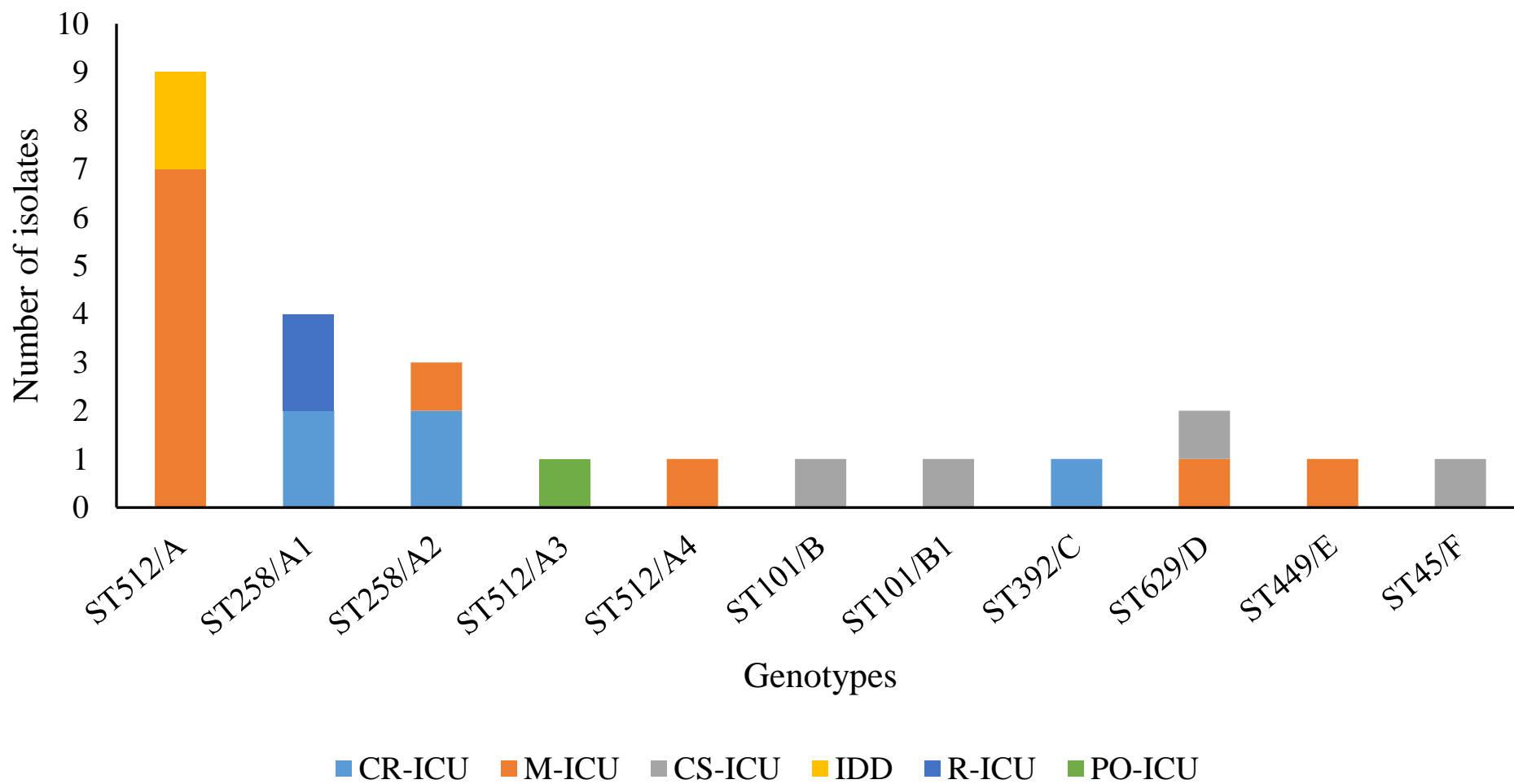

Supplement: Figure S1 — Circulation of colistin-resistant K. pneumoniae genotypes in the HA “Ospedale dei Colli” from January 2015 to September 2016. The number of isolates and wards are shown. [file Image_1.PDF]

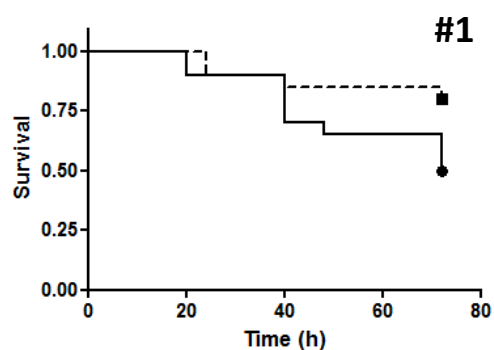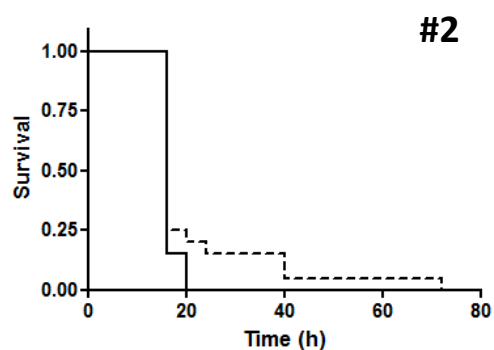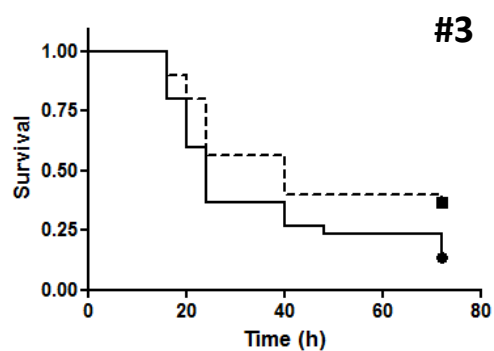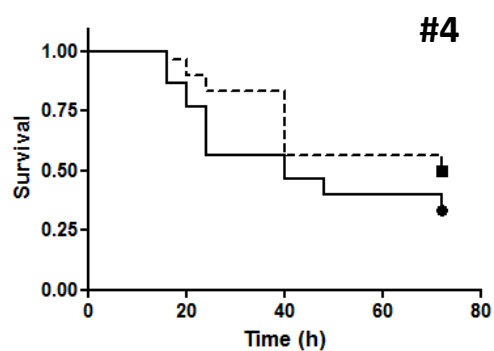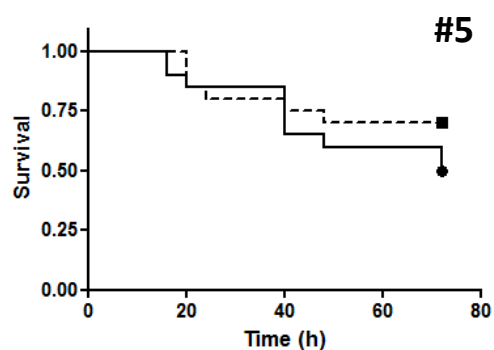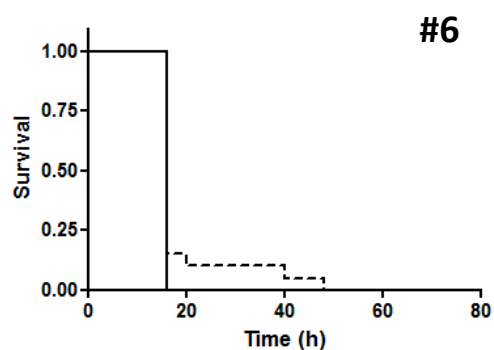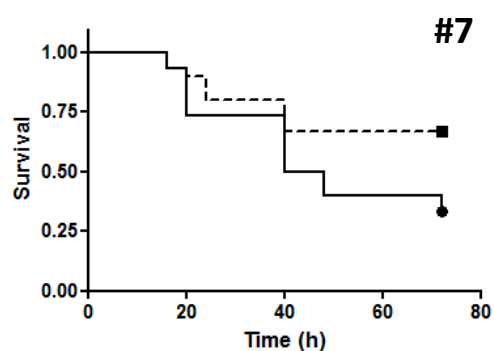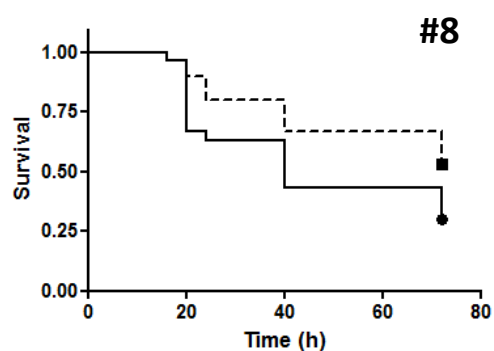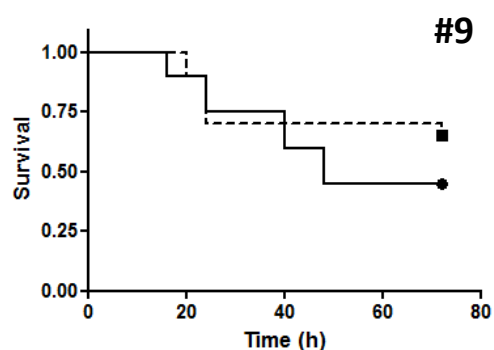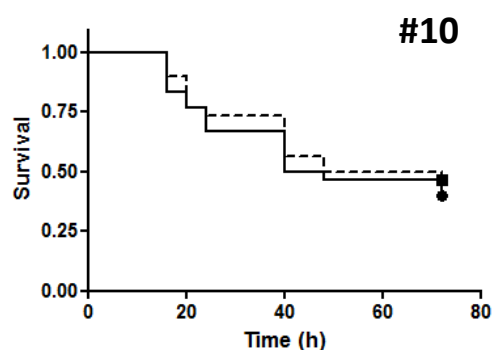

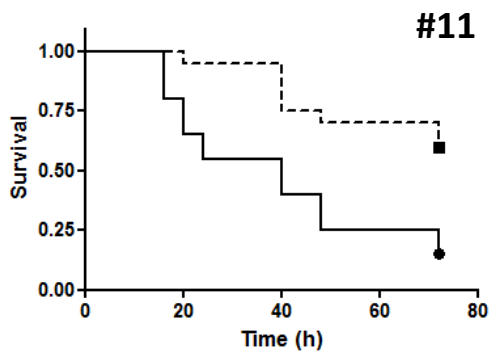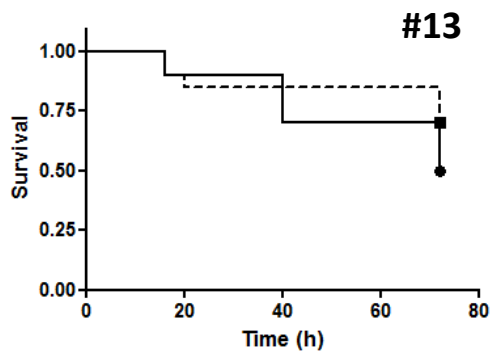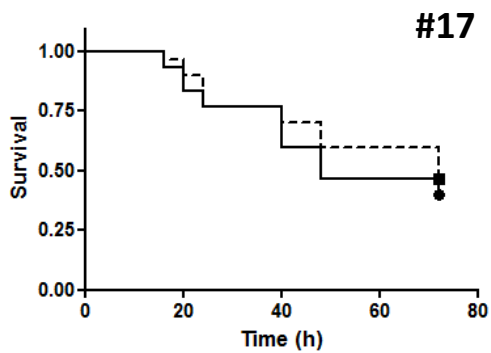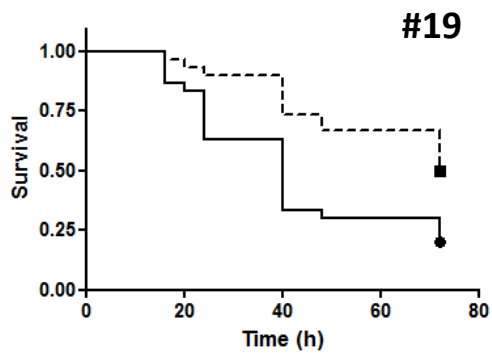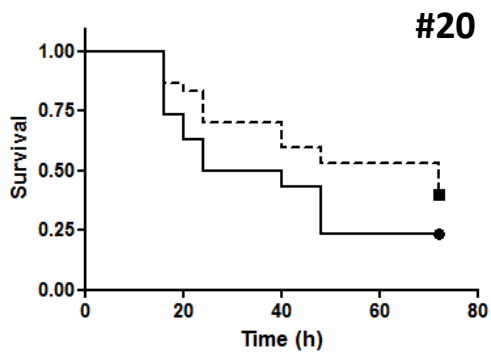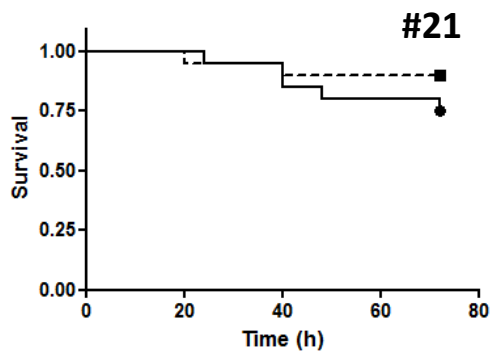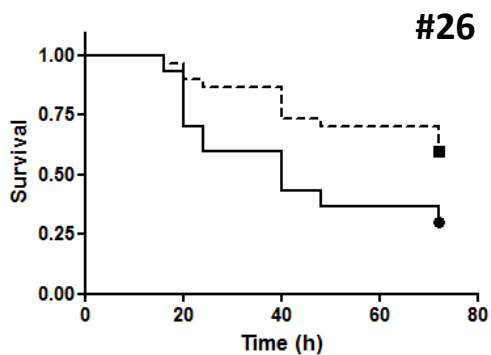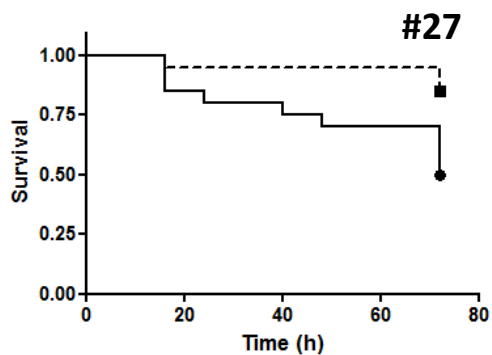

Supplement: Figure S2 — Kaplan–Meier survival curves of G. mellonella larvae infected with 1 (±0.26) × 106 (continuous line) or 1 (±0.26) × 105 (dashed line) viable cells of the indicated K. pneumoniae strains. For each strain, 20 or 30 larvae were infected in two or three independent assays, respectively. In both experiments, the infectivity of strains #2 and 6 is significantly higher than that of all other strains (P < 0.001). [file Image_2.PDF]

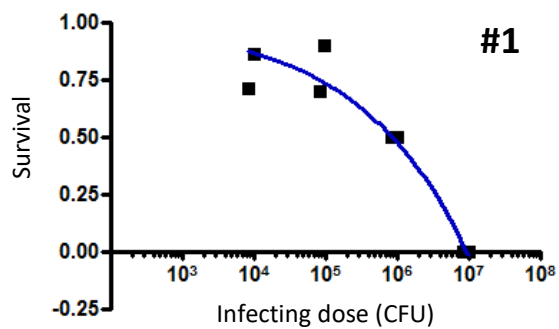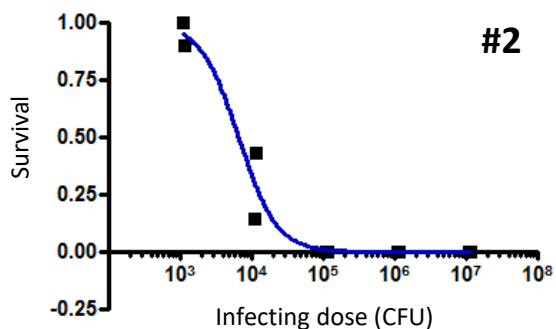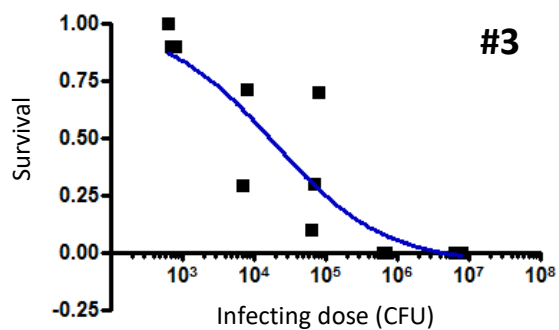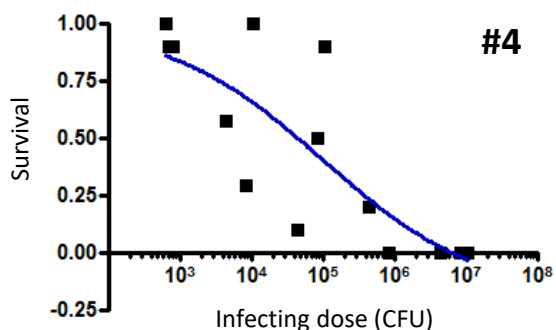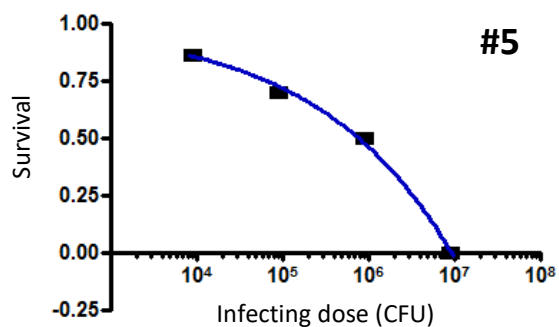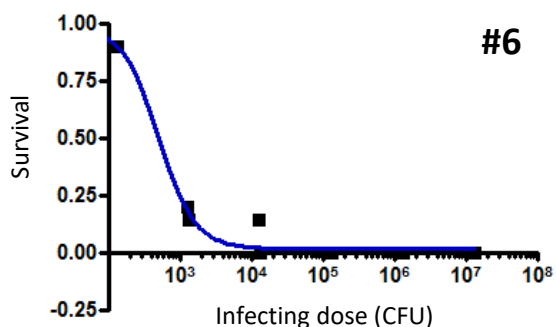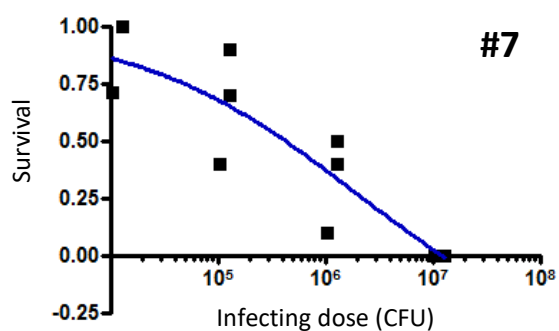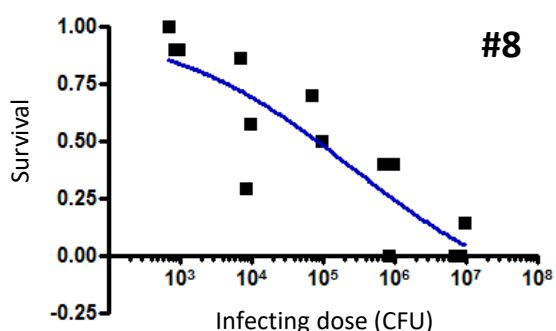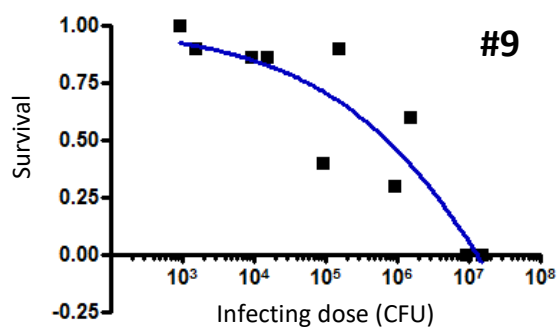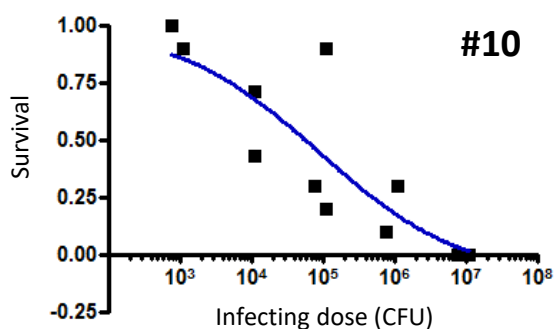

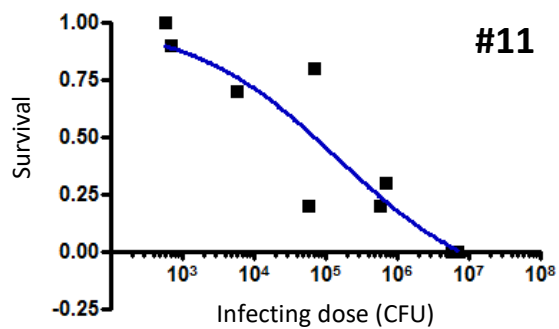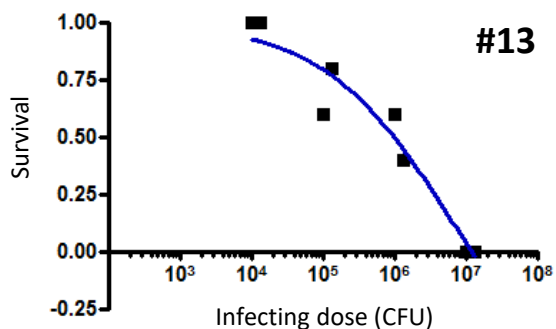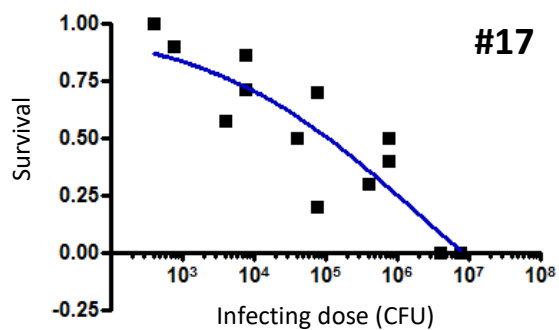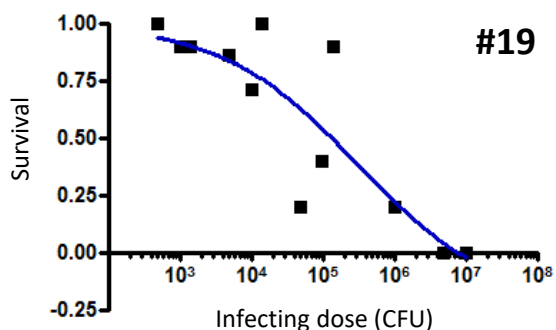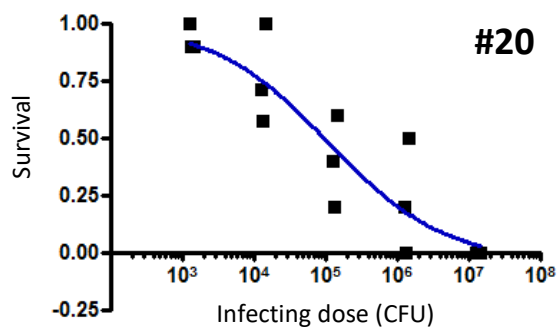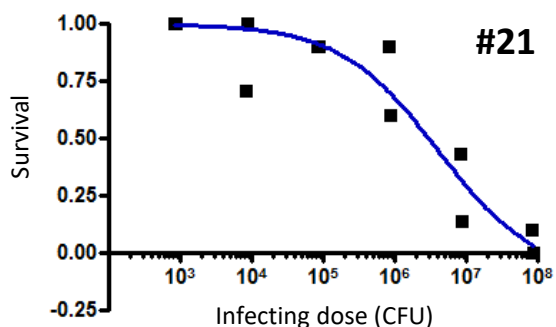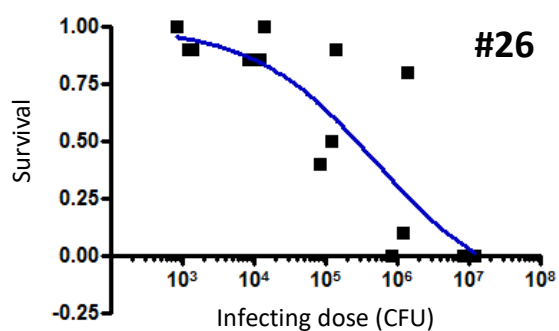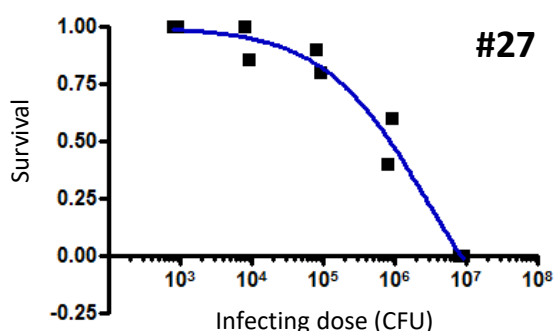

Supplement: Figure S3 — Dose-dependent survival curves, generated by the GraphPad Prism software, of G. mellonella larvae infected with different doses of the K. pneumoniae strains #1, 2, 3, 4, 5, 6, 7, 8, 9, 10, 11, 13, 17, 19, 20, 21, 26, and 27. These curves have been used to retrieve the LD50, LD90, and R2 values reported in Table 4. [file Image_3.PDF]
