# Supplementary material for: Molecular Epidemiology and Virulence Profiles of Colistin-Resistant Klebsiella pneumoniae Blood Isolates From the Hospital Agency “Ospedale dei Colli,” Naples, Italy
Source: Front Microbiol. 2018 Jul 16;9:1463. doi: 10.3389/fmicb.2018.01463 (PMC6054975; doi:10.3389/fmicb.2018.01463)
Supplement: Table S1 — Oligonucleotides used in the study. [file Table_1.DOCX]

**Table S1**. Oligonucleotides used in the study.

| Primer | Sequence (5’-3’) | Purpose | Reference |
| --- | --- | --- | --- |
| CLR F | CGGTCAGTCCGTTTGTTC | *mcr-1* PCR | Liu et al., 2015 |
| CLR R | CTTGGTCGGTCTGTAGGG | *mcr-1* PCR | Liu et al., 2015 |
| MCR2 IF | TGTTGCTTGTGCCGATTGGA | *mcr-2* PCR | Xavier et al., 2016 |
| MCR2 IR | AGATGGTATTGTTGGTTGCTG | *mcr-2* PCR | Xavier et al., 2016 |
| MCR3-F | TTGGCACTGTATTTTGCATTT | *mcr-3* PCR | Yin et al., 2017 |
| MCR3-R | TTAACGAAATTGGCTGGAACA | *mcr-3* PCR | Yin et al., 2017 |
| Mcr-4 FW | ATTGGGATAGTCGCCTTTTT | *mcr-4* PCR | Carattoli et al., 2017 |
| Mcr-4 RV | TTACAGCCAGAATCATTATCA | *mcr-4* PCR | Carattoli et al., 2017 |
| mgrB_ext_F | AAGGCGTTCATTCTACCACC | *mgrB* PCR | Cannatelli et al., 2013 |
| mgrB_ext_R | TTAAGAAGGCCGTGCTATCC | *mgrB* PCR | Cannatelli et al., 2013 |
| Int_mgrB_F | CGGTGGGTTTTACTGATAGTCA | *mgrB* sequencing | Cannatelli et al., 2014 |
| Int_mgrB_R | ATAGTGCAAATGCCGCTGA | *mgrB* sequencing | Cannatelli et al., 2014 |
| pmrA_ext_F | CATTTCCGCGCACTGTCTGC | *pmrA* PCR and sequencing | This study |
| pmrA_ext_R | CAGGTTTCAGTTGCAAACAG | *pmrA* PCR and sequencing | This study |
| pmrB_Ext14_F | CAACTGAAACCTGGACCATGC | *pmrB* PCR | This study |
| pmrB_Ext1097_R | CAATGGGTGCTGACGTTCTG | *pmrB* PCR | This study |
| pmrB_Int177_F | GGAGCATGAGGTACGGGAAG | *pmrB* sequencing | This study |
| pmrB_Int797_R | GCATTCTCCACCAGATTGCG | *pmrB* sequencing | This study |
| pmrB_Int_878_R | TCATCTTCCACCGCCATCAC | *pmrB* sequencing | This study |
| CrrB-For_Ext | AGACTCGCTGGATCGTACTG | *crrB* PCR | This study |
| CrrB-Rev_Ext | CTCGGCAGTATGTGGGATCT | *crrB* PCR | This study |
| CrrB-Rev_Int964 | ACAACCCTAAACCACAGCCC | *crrB* sequencing | This study |
| CrrB-Rev_Int680 | TCAAAGTCCGGCCAAAAGGT | *crrB* sequencing | This study |
| CrrB-Rev_Int481 | CCTGAAGCCTCCCACGTAAG | *crrB* sequencing | This study |
| phoP ext F | GAGCKTCAGACTACTATCGA | *phoP* PCR | This study |
| phoP ext R | GGGAAGATATGCCGCAACAG | *phoP* PCR | Jayol et al., 2014 |
| phoP int F | GCGTCACCACCTCAAAGTTC | *phoP* sequencing | Jayol et al., 2015 |
| phoP int R | GGCGATATCCGGGAGATGTT | *phoP* sequencing | Jayol et al., 2015 |
| phoQ ext F | GAATACCCACAGGACGTCAT | *phoQ* PCR | This study |
| phoQ ext R | CAGGTGTCTGACAGGGATTA | *phoQ* PCR | Jayol et al., 2014 |
| phoQ int F | CTCAAGCGCAGCTATATGGT | *phoQ* sequencing | Jayol et al., 2015 |
| phoQ int R | TCTTTGGCCAGCGACTCAAT | *phoQ* sequencing | Jayol et al., 2015 |
| phoQ F2 | GCATCTCGCAGCAGATTGGT | *phoQ* sequencing | This study |
